# Wake Detection and Positioning for Autonomous Underwater Vehicles Based on Cilium-Inspired Wake Sensor

**DOI:** 10.3390/s25010041

**Published:** 2024-12-25

**Authors:** Xuanye Hu, Yi Yang, Zhiyu Liao, Xinghua Zhu, Renxin Wang, Peng Zhang, Zhiqiang Hu

**Affiliations:** 1 State Key Laboratory of Robotics, Shenyang Institute of Automation, Chinese Academy of Sciences, Shenyang 110016, China; huxuanye@sia.cn (X.H.); liaozhiyu@sia.cn (Z.L.); zhuxinghua@sia.cn (X.Z.); hzq@sia.cn (Z.H.); 2 University of Chinese Academy of Sciences, Beijing 100049, China; 3 State Key Laboratory of Dynamic Measurement Technology, North University of China, Taiyuan 030051, China; wangrenxin@nuc.edu.cn (R.W.); s202206014@st.nuc.edu.cn (P.Z.)

**Keywords:** cilium-inspired wake sensor, wake detection, wake tracking, target positioning, autonomous underwater vehicles

## Abstract

This paper proposes a method for passive detection of autonomous underwater vehicle (AUV) wakes using a cilium-inspired wake sensor (CIWS), which can be used for the detection and tracking of AUVs. First, the characteristics of the CIWS and its working principle for detecting underwater flow fields are introduced. Then, a flow velocity sensor is used to measure the flow velocities of the “TS MINI” AUV’s wake at different positions, and a velocity field model of the “TS MINI” AUV’s wake is established. Finally, the wake field of the “TS MINI” AUV was measured at various positions using the CIWS. By analyzing the data, the characteristic frequency of the AUV’s propeller is identified, which is correlated with the AUV’s rotation speed and the number of blades. Through further analysis, a mapping model is established between the spectral amplitude of the characteristic frequency at different positions and the corresponding wake velocity. By substituting this mapping model into the AUV’s wake velocity field model, the possible position range of the sensor relative to the AUV propeller can be estimated. The research provides a technical foundation for underwater detection and tracking missions based on wake detection.

## 1. Introduction

Autonomous underwater vehicles (AUVs) offer several advantages, including operational flexibility, strong stealth capability, adaptability to complex ocean conditions, and cost-effectiveness. With the continuous development of AUV technology, the complexity and difficulty of their missions have also increased significantly, making a single AUV insufficient for meeting increasingly complex demands [1]. This has led to the inevitable trend of multiple AUVs collaborating in swarms to carry out new missions [2,3,4]. AUV swarms are similar to the fish swarms. Traditionally, AUV swarms use acoustic or visual communication or position methods [5,6,7,8]. However, fish rely primarily on their highly sensitive lateral line organs to sense the surrounding fluid environment, allowing them to perform essential tasks like foraging and obstacle avoidance. Inspired by biomimetics, sensors with functions similar to lateral lines can be used to detect the wakes produced by other AUVs during navigation, providing AUVs with sensory capabilities comparable to those of fish. This allows AUVs to discern other weak moving targets underwater and enables the AUV swarm formation similar to fish schooling. Lateral line sensing, compared to traditional sonar and visual detection methods, features a simpler device structure. As a passive detection technology, it offers the advantage of lower energy consumption compared to active sonar and underwater cameras. In complex underwater environments, sonar is prone to interference from surrounding objects, while visual detection relies on adequate lighting and high water clarity. In such harsh hydrological conditions, lateral line sensing technology remains effective and reliable.

Bio-inspired sensor technology has become a significant research area in recent years [9,10]. Astreinidi’s [11] research explored how biological organisms use mechanical interactions to acquire information and assessed the feasibility of applying these principles to mechanical design. Sietse M. van Netten [12,13] validated the fish lateral line dynamics model through calculations and experiments and elucidated the signal-processing capabilities of the lateral line canal organ by studying its frequency response, which had proven its capability to maintain constant sensitivity to fluid acceleration at low frequencies. Debarun Sengupta [14] proposed a novel fabrication process for fabricating inexpensive yet sensitive bio-inspired cilium sensors and demonstrated their potential applications in flow velocity detection. Qiao [15] encapsulated and adapted the cilium-inspired wake sensors, making them feasible for AUV wake detection.

However, currently, no researchers have practically applied CIWSs for AUV wake detection or further AUV swarm perceptions. One of the application scenarios for the existing wake detection technology in underwater vehicles is torpedo wake guidance [16]. Using CIWSs can determine the relationship between wake velocity and sensor signal amplitude, offering higher measurement accuracy than traditional wake guidance and enabling effective underwater target detection and tracking. Unlike conventional torpedo wake guidance, which requires frequent traversals across the wake boundary to sense the wake [17,18,19], CIWSs can perceive the wake continuously within its range, thereby effectively reducing energy consumption during the tracking process.

In this paper, the working principle of the CIWS is analyzed and the relationship between the wake velocity and the sensor signal amplitude is established. Subsequently, the spectral data of the sensor at different positions in the wake are analyzed, and the correlation between the sensor data and the relative position in the wake is established through a wake decay model, all of which provides a theoretical foundation for subsequent wake perception and target tracking based on CIWSs.

## 2. CIWS Model

### 2.1. Flow Field Sensing Principle Based on Bio-Inspired Cilium

**Figure 1:**
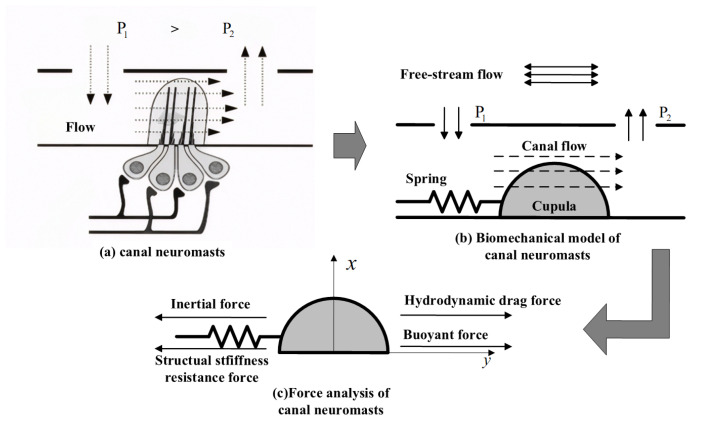
Schematic diagram of the biomechanics of the CN [20]. (**a**) Structural components of the CN. (**b**) Biomechanical model of the CN. (**c**) Force analysis of the CN.

The lateral line system is a specialized mechanosensory system found in fish and certain amphibians. The lateral line sensory system in fish consists of superficial neuromasts and canal neuromasts(CN), both of which rely on cilium-based receptors to perform their primary functions [20]. The superficial neuromasts are positioned on the surface of the fish, directly exposed to the external flow, and contribute to flow sensing. The CNs are embedded beneath the epidermis within the lateral line canal and connect to the external water flow through small pores on the fish’s skin, thereby enabling the detection of pressure fields in the water. The lateral line canal effectively filters out low-frequency noise generated by the fish’s own movement, as well as other environmental noise, thereby enhancing the fish’s ability to detect targets. The biomechanical model of the CN can be represented as a rigid hemisphere sliding without friction on a plane. This rigid hemisphere is coupled to a linear spring, with its stiffness provided by the cilium, as shown in Figure 1.

Figure 1c shows the forces on the cupula of CN.Taking the hemisphere as the main body, the mechanical control equation of the CN in the lateral line system is defined as

(1) $$F_{i}+F_{s}=F_{h}+F_{b}$$

where $F_{i}$ represents the inertial force of the hemisphere, which can be expressed as

(2) $$F_{i}=M\frac{d^{2}Y\left(t\right)}{dt^{2}}$$

Here, *M* is the mass of the hemisphere and $M=4\pi a^{3}\rho /3$, *a* is the radius of the hemisphere, ρ is the density of the hemisphere. Y(t) is the displacement of the hemisphere induced by the fluid flow, where $Y\left(t\right)=Y_{0}\left(f\right)\exp\left(i2\pi ft\right)$ is the steady-state oscillatory displacement. $Y_{0}\left(f\right)$ represents the displacement amplitude of the hemisphere, which is a function of the stimulus frequency *f*.

In Equation (1), $F_{s}$ represents the structural stiffness force due to the elastic coupling between the hemisphere and the epithelial cells, which can be defined as

(3) $$F_{s}=KY\left(t\right)$$

where *K* is the sliding coefficient, which is proportional to the number of epithelial cells.

In Equation (1), the first term on the right-hand side, $F_{h}$, represents the hydrodynamic drag force acting on the hemisphere, which can be defined as

(4) $$F_{h}=D\left(\frac{dY(t)}{dt}-\frac{dW(t)}{dt}\right)$$

where *D* is the drag coefficient, and dY(t)/dt−dW(t)/dt represents the relative displacement between the hemisphere and the external flow velocity. $W\left(t\right)=W_{0}\left(f\right)\exp\left(i2\pi ft\right)$ represents the oscillatory displacement of the external flow. $W_{0}\left(f\right)$ is the amplitude of the external fluid displacement, and *f* is the excitation frequency.

In Equation (1), $F_{b}$ represents the buoyancy force acting on the hemisphere due to the pressure difference around the structure, which can be defined as

(5) $$F_{b}=M\frac{d^{2}W\left(t\right)}{dt^{2}}$$

Thus, the mechanical control equation can also be expressed as

(6) $$M\frac{d^{2}Y\left(t\right)}{dt^{2}}+KY\left(t\right)=D\left(\frac{dY(t)}{dt}-\frac{dW(t)}{dt}\right)+M\frac{d^{2}W\left(t\right)}{dt^{2}}$$

The control equation is linear and can be solved in the frequency domain. Therefore, by solving this control equation, the frequency-dependent sensitivity of the hemisphere can be obtained. Sensitivity is defined as the ratio of the displacement amplitude $Y_{0}\left(f\right)$ to the external fluid velocity amplitude $V_{0}\left(f\right)$. The external fluid velocity term can be obtained from Equation (6):

(7) $$V\left(t\right)=\frac{dW(t)}{dt}=i2\pi fW_{0}\left(f\right)\exp\left(i2\pi ft\right)=V_{0}\left(f\right)\exp\left(i2\pi ft\right)$$

Thus, the frequency-dependent sensitivity of the hemisphere can be expressed as

(8) $$S_{cn}\left(f\right)=\frac{Y_{0}\left(f\right)}{V_{0}\left(f\right)}=\frac{1}{2\pi f_{t}}\frac{1+\frac{\sqrt{2}}{2}\left(1+i\right){\left(\frac{f}{f_{t}}\right)}^{1/2}+\frac{1}{3}i\frac{f}{f_{t}}}{N_{r}+i\frac{f}{f_{t}}-\frac{\sqrt{2}}{2}\left(1+i\right){\left(\frac{f}{f_{t}}\right)}^{3/2}-\frac{1}{3}i{\left(\frac{f}{f_{t}}\right)}^{2}}$$

where $f_{t}=\mu /2\pi \rho_{w}a^{2}$ is the transition frequency at which either viscous force ($f<f_{t}$) or inertial force ($f>f_{t}$) acts on the hemisphere. μ is the dynamic viscosity of the fluid. $N_{r}=Ka\rho_{w}/6\pi \mu^{2}$ is the resonance factor affecting the resonance of the hemisphere, and $\rho_{w}$ is the density of water. The frequency response model can be represented by a transfer function. The transfer function is defined as the ratio of the stimulus variable to the response variable and can therefore be used as a mathematical expression for sensitivity. The frequency response of the hemisphere varies with the frequency of the external flow stimulation.

The entire process of external flow excitation acting on the hemisphere can be divided into two steps. In the first step, the velocity or acceleration of the external free flow is transformed by the canal into a local flow velocity. The external flow generates a pressure difference between the lumen, which, in turn, creates a local flow velocity in the canal. In this step, sensitivity or the transfer function is defined as the ratio of the local flow velocity to the free flow velocity, denoted as $S_{cn_{0}}\left(f\right)$. $S_{cn_{0}}\left(f\right)$ can be analytically solved using basic hydrodynamics theory and cylindrical flow theory. In the second step, the flow inside the canal generates fluid forces acting on the hemisphere, causing it to slide. The sensitivity $S_{cn}\left(f\right)$ is defined as the ratio of the displacement amplitude of the hemisphere to the flow velocity inside the canal, as shown in Equation (8). By calculating the product of the above two transfer functions, the composite frequency response of the hemisphere can be obtained, which provides the relationship between the hemisphere displacement and the external canal flow velocity.

The excitation of the hemisphere by the external water flow can be divided into the following two steps. In the first step, the speed or acceleration of the free external flow is transformed by the canal into the local flow speed. The external flow creates a pressure difference across the canal, which, in turn, generates a local flow velocity within the canal. In this step, the sensitivity or transfer function is defined as the ratio of the local flow speed to the free flow speed, which can be analytically solved using basic hydrodynamic theory and cylindrical flow theory. In the second step, the fluid flow within the canal generates a fluid force acting on the hemisphere, causing the hemisphere to slide. The sensitivity is defined as the ratio of the displacement amplitude of the hemisphere to the velocity of the flow within the canal, as shown in Equation (8). By calculating the product of these two transfer functions, the composite frequency response of the hemisphere can be obtained. Using this composite frequency response, the relationship between the displacement of the hemisphere and the external flow velocity can be determined.

### 2.2. CIWS

**Figure 2:**
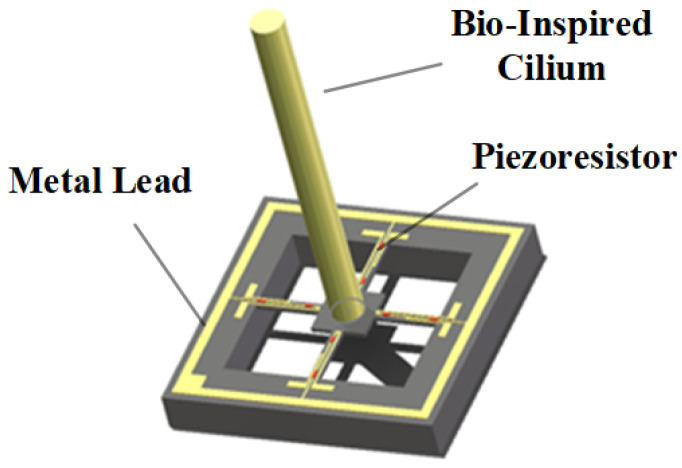
Cilium-Crossbeam Stress and Voltage Output Model for CIWS.

In this study, the CIWS independently developed by North University of China was selected [21]. The sensor utilizes a cilium-crossbeam stress and voltage output model, as shown in Figure 2. The model employs cylindrical cilium, with piezoresistive elements distributed at the points of maximum stress on the intersecting cantilever beams, thereby maximizing the sensitivity of the sensing units. When different flow velocities act on the cilium, the cantilever beam deforms in response to the direction of the flow velocity. This deformation changes the resistance of the piezoresistors, which can be measured by monitoring the voltage across different piezoresistors.

**Figure 3:**
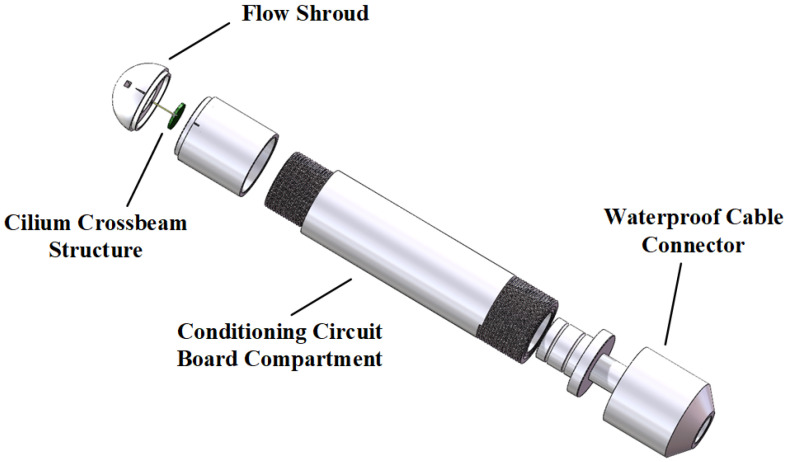
Structure of the CIWS.

The packaging of the CIWS is shown in Figure 3. An underwater fairing featuring four evenly distributed holes around its structure is installed at the head of the sensor, directing water flow onto the cilium-crossbeam structure. The underwater fairing is mounted at the sensor head, and the deformation of the cilium generates a potential difference, which is transmitted to a conditioning circuit board to amplify the voltage in each signal channel. The amplified signals are transmitted to the signal acquisition circuit through waterproof cables. The specific parameters of the CIWS are listed in Table 1.

**Figure 4:**
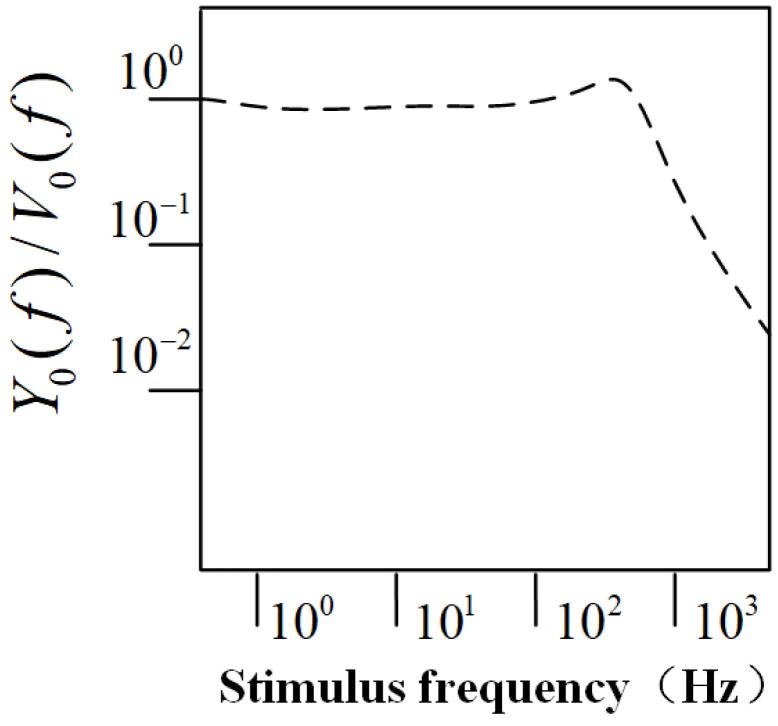
The frequency response curve as a function of the excitation frequency [21].

From Equation (7), it can be derived that the sensitivity of the CIWS is defined as the ratio of the hemispherical displacement amplitude $Y_{0}\left(f\right)$ to the external flow velocity amplitude $V_{0}\left(f\right)$. The frequency response curve as a function of the excitation frequency is shown in Figure 4. From the figure, it can be observed that the ratio of the hemispherical displacement to the external flow velocity amplitude fluctuates around $10^{0}$ in the low-frequency range, indicating that the hemispherical displacement is approximately proportional to the external flow velocity. Furthermore, based on the elastic mechanics formula: F=kx, it follows that the hemispherical displacement is directly proportional to the force acting on the hemisphere. Therefore, it can be concluded that the force acting on the hemisphere is linearly related to the external flow velocity.

The displacement of the hemisphere is approximately proportional to the external flow velocity in the low-frequency range, meaning that the CIWS data can intuitively reflect the flow velocity at the current position. However, in addition to the vortices, the wake also contains turbulent flows, resulting in unstable local flow velocities. This leads to large fluctuations in the corresponding time-domain signal, without any distinct signal features, making typical feature matching algorithms unsuitable for time-domain analysis. Calculating the average wake velocity over a certain period can represent the average velocity at the current position. However, during AUV navigation, it is not possible for the AUV to remain at the same position for an extended period, necessitating real-time acquisition of wake velocity information. Therefore, using spectral analysis to obtain wake velocity is a feasible approach. As previously analyzed, in the low-frequency domain, the displacement of the hemisphere is linearly related to the external flow velocity. Thus, in the low-frequency part of the spectrum shown in Figure 4, it is possible to identify frequency points where the displacement of the hemisphere is linearly related to the external flow velocity. The spectral amplitude at these frequency points can then be used to reflect the flow velocity at the current position.

## 3. Experimental Testing and Modeling of the Flow Field of Pump-Jet Propellers

The sensitivity of the CIWS tends to stabilize at a constant value in the low-frequency range. The voltage of the sensor corresponds to the displacement of the cilium, and the displacement of the cilium is linearly related to the external flow velocity. To quantitatively analyze the relationship between the CIWS signal and the wake velocity at the current position, it is necessary to acquire the CIWS data at different positions in the wake velocity field, along with the corresponding flow velocity values.

### 3.1. Wake Field Generation Using Pump-Jet Propellers

**Figure 5:**
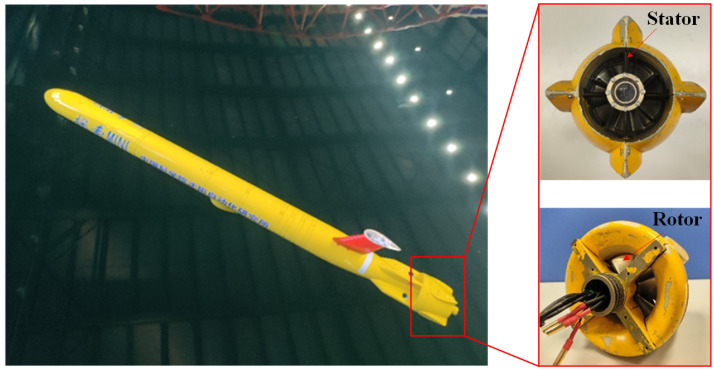
Photograph of the Aft Stator Pump-Jet Propeller.

In this study, the “TS MINI” AUV [22], developed by the Shenyang Institute of Automation, Chinese Academy of Sciences, was selected to generate the AUV wake, as shown in Figure 5. The propulsion system is a pump-jet propeller (PJP) with an aft stator. The specific parameters of the PJP are listed in Table 2.

**Figure 6:**
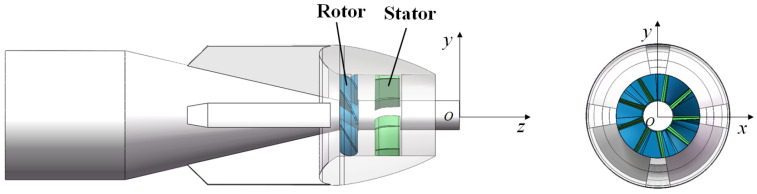
The PJP Model and Reference Coordinate System.

To standardize the orientation of the coordinate axes and the position of the origin for subsequent analysis, an inertial reference coordinate system *O*-*xyz* is established based on the PJP model, as shown in Figure 6. The origin *O* is located at the center point of the plane at the downstream end of the PJP rotational axis. The *y*-axis points vertically upward, the *z*-axis is aligned with the central axis and points towards the outlet, and the *x*-axis follows the right-hand rule. Here, *n* represents the rotation speed of the rotor (with the direction of rotation defined by the right-hand rule).

### 3.2. Wake Flow Field Measurement

The PJP wake comprises both turbulence and vortices, leading to fluctuations in the velocity of the wake field. To determine the average velocity at a given position, the flow velocity at a fixed position was measured over a specific period of time, and the mean value was calculated. In this experiment, the wake was generated using the “TS MINI” AUV PJP. The experimental setup consisted of a tank with dimensions of 4 m in length, 2 m in width, and 1 m in height with a water depth of 68 cm. The PJP was mounted on a frame positioned 20 cm above the bottom of the tank. A 1m×2m frame was placed behind the PJP to hold the sensors, and a ruler was attached to the frame to measure the exact coordinates of the sensor.During the experiment, the PJP operated in a moored state, and the selected AUV had a relatively low cruising speed, ensuring that the propeller wake dominated the flow field. The wake flow field under moored conditions can effectively reflect the wake characteristics during navigation. Additionally, the PJP exhibits strong jet-focused characteristics, resulting in slower lateral wake diffusion. The impact of echo effects from the wake reaching the tank walls can be considered negligible. Moreover, the length of the tank is significantly greater than the extent of the wake, rendering the axial echo effects negligible as well.

**Figure 7:**
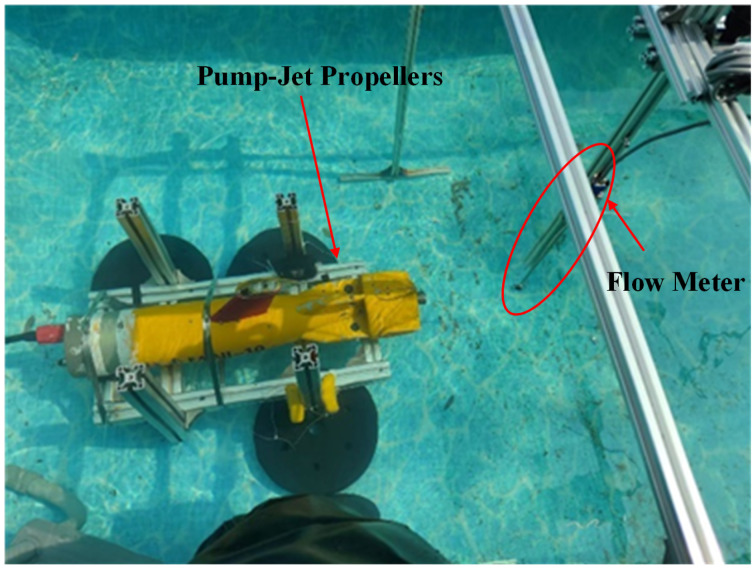
Measurement Site for the Average Velocity of PJP Wake.

The LS300A flow meter, manufactured by Nanjing Ouka Instrument and Meter Co., Ltd. (Nanjing, China), was utilized for velocity measurements, maintaining the flow meter at the same height as the propeller. Measurements were conducted along the Oz axis, with the *x*-axis ranging from −10 cm to 10cm and the *z*-axis ranging from 10cm to 110cm, resulting in 11×56 velocity measurement points. The PJP rotational speed was set to 1000 rpm (actual speed of 960 rpm), which corresponds to one of the operating speeds of the “TS MINI” AUV, with a cruising speed of 3 knots. At this speed, the wake field intensity is suitable for measurement and provides a rapid response for AUV navigation control. The flow meter measured the flow velocity at each position for one minute, and the average value was calculated to represent the mean velocity at each position. Measurements were repeated three times for each position. The experimental setup is shown in Figure 7.

**Figure 8:**
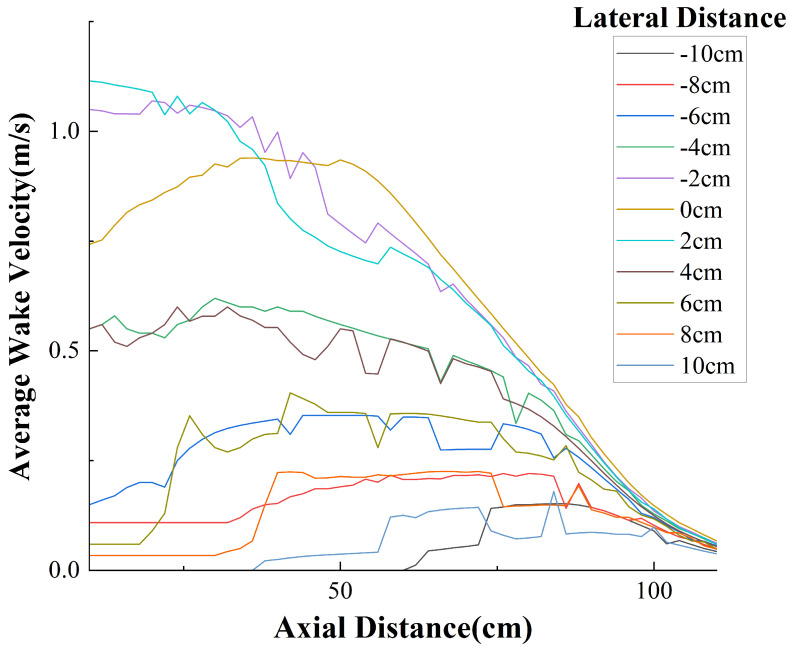
Relationship Between Measured Wake Velocity and Position.

**Figure 9:**
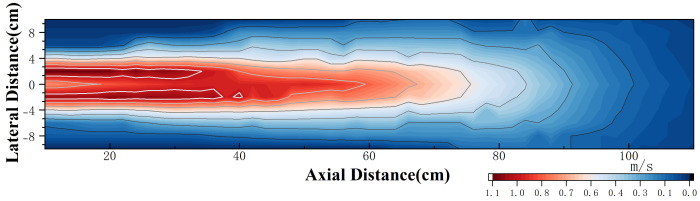
Iso−Velocity Contour Map of the Wake Flow Field.

The experimental measurement data are presented in Figure 8. It can be observed that due to the obstruction caused by the central shaft of the PJP, the velocity curve at a lateral offset of 0cm is not the maximum, and the axial velocity at this point initially increases and then decreases. The data for other lateral offsets generally follow a decaying trend. The average velocity demonstrates an approximately symmetrical relationship across the lateral distances. The velocity field of the PJP is shown in Figure 9. From the figure, it can be observed that the maximum flow velocity is approximately 1.1m/s. When the axial distance exceeds 100cm, the wake velocity tends to stabilize. Additionally, beyond a lateral distance of 10cm, the wake flow is essentially negligible.

## 4. Wake Detection Experiment Based on CIWS

In the previous section, an average velocity decay model of the wake was established based on the measurement of average velocity within the wake region of the propeller. To further investigate the relationship between the wake sensor signals and the average wake velocity, it is necessary to measure the wake sensor data under the same operating conditions within the PJP’s wake region. The wake sensor utilized is a cilium-based MEMS wake sensor developed by North University of China, with the wake still generated by the “TS MINI” AUV PJP.

**Figure 10:**
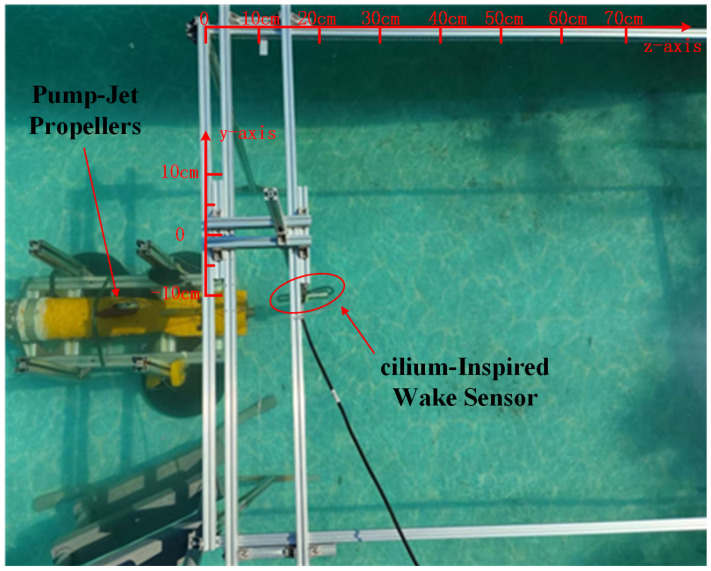
Data Collection Site of the CIWS.

The PJP and sensor were placed in a tank with dimensions of 4m in length, 2m in width, and 1m in height, with a water depth of 68cm. The wake data from the “TS MINI” AUV were collected using the wake sensor. The PJP was fixed onto a frame positioned 20cm above the bottom of the tank, while a 1m×2m frame was placed behind it to fix the sensor. The frame was equipped with rulers along the *z*-axis and *y*-axis to mark the sensor’s specific coordinates. The *z*-axis measurement range started at 10cm and extended to 100cm, while the effective measurement range along the *y*-axis spanned from −10cm to 10cm. This experiment was conducted under static conditions, with both the PJP and the sensor held in fixed positions. The PJP was positioned at the center of the tank, equidistant from both sides, and the sensor was used to collect data at predefined distances. The experimental setup is illustrated in Figure 10.

**Figure 11:**
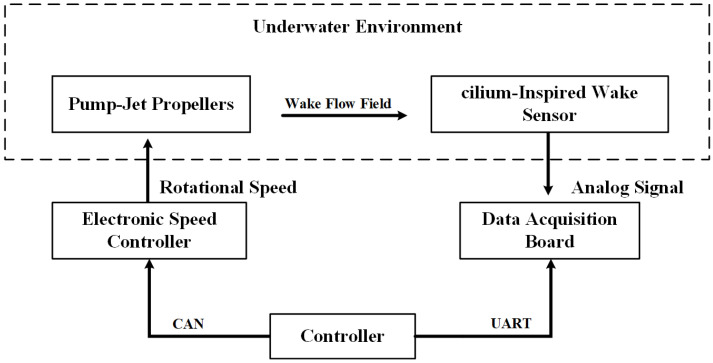
Block Diagram of the Wake Data Acquisition Experimental Setup.

The PJP rotation speed was set to 1000rpm (actual speed of 960rpm). Under these conditions, measurements were conducted for axial data along the positive *z*-axis at an *x*-axis offset of 0, and for lateral data along the *x*-axis at a *z*-axis offset of 30cm. Specifically, for the axial data, measurements were taken from 10cm to 100cm at 10cm intervals, resulting in 10 data points. For the lateral data, measurements were taken from −10 cm to 10cm at 2cm intervals, resulting in 11 data points. Throughout the entire measurement process, the sensor was aligned parallel to the longitudinal axis.The block diagram of the wake data acquisition experimental setup is shown in Figure 11.

The CIWS used was a cilium-crossbeam structure, which has two-dimensional sensing capability. To simplify the experiment, the *y*-axis offset was kept at 0 throughout the measurements, thereby enabling the assessment of the sensor’s sensitivity in a single dimension. This experimental setup is consistent with the fixed-depth navigation mode of an AUV during underwater operations.

Data collection was conducted using a signal acquisition circuit based on the WM8978 chip from Wolfson Microelectronics. The deformation of the cilium caused a change in the resistance of the piezoresistor, generating a potential difference. The signal acquisition circuit converted this voltage into digital signals through analog-to-digital conversion (ADC) and recorded the data, with a sampling frequency of 96kHz. Given that the sensitivity of the CIWS remains stable for low-frequency components, this study focused on the low-frequency portion of the signal spectrum, for which the chosen sampling frequency was sufficient. The duration of each sampling session was 10s, and each measurement point was sampled six times to minimize random error. During the entire measurement process, the PJP remained continuously operational. After relocating the sensor to the next measurement point, data collection commenced following a waiting period of 1min to reduce errors associated with movement.

**Figure 12:**
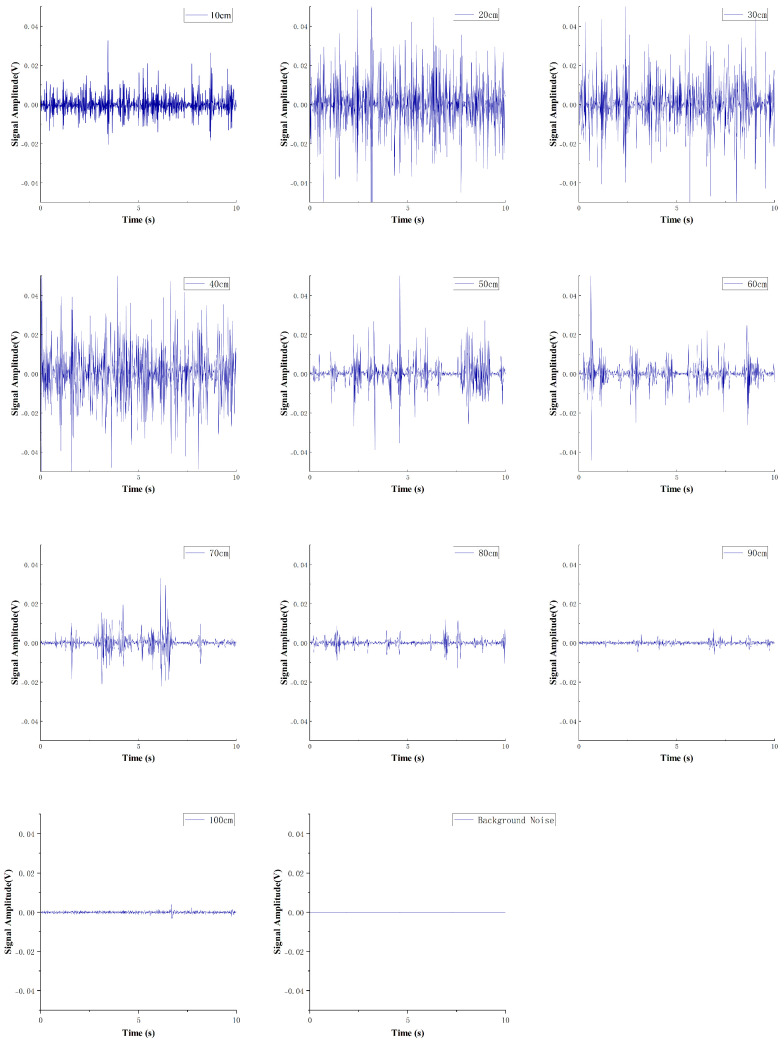
Time −Domain Signals Measured by the CIWS at Different Axial Distances.

**Figure 13:**
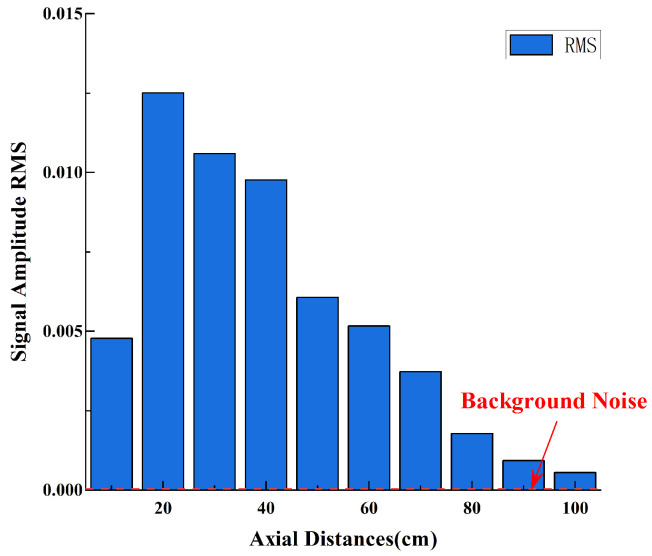
Time−Domain Signal Amplitude RMS at Different Axial Distances.

The final experiment produced time-domain data for the wake across a 90cm×20cm area, including one set of axial distance data and one set of lateral distance data. Each sampling point was measured six times, resulting in a total of 21 sets of 126 time-domain data samples. Figure 12 presents ten sets of time-domain signals collected by the wake sensor at an *x*-axis offset of 0, with the *z*-axis offset ranging from 10cm to 100cm, under the PJP operating condition of 960rpm, as well as one set of background noise signals with the PJP not working. It can be observed from the figure that the signal intensity at 10cm is not the highest due to the obstruction of the PJP central shaft. As the *z*-axis offset increases, the signal intensity initially increases and then decreases. Specifically, at distances of 90cm and 100cm from the propeller, the signals detected by the sensor are weak and difficult to distinguish. However, the signals at both locations are still significantly higher than the background noise while the propeller is not in operation. This indicates that the cilium-based sensor has greater sensitivity to the propeller’s noise compared to flow velocity. The root mean square (RMS) values of the time-domain signal amplitude shown in Figure 13 corroborate the aforementioned trend, where the signal strength initially increases and subsequently decreases with increasing axial distance.

The time-domain data from the CIWS can intuitively reflect the flow velocity at the current position. However, due to the turbulent nature of the wake, the flow is complex and difficult to predict, and as shown in Figure 12, there are no distinctive features in the time-domain signal. Therefore, subsequent analysis can be conducted through spectral analysis to determine the relationship between the spectral amplitude and the average wake velocity. Since spectral data can be obtained in real-time, the results of the spectral analysis can effectively guide the wake detection process of the AUV.

## 5. Wake Data Analysis and Positioning

### 5.1. Frequency Domain Analysis

**Figure 14:**
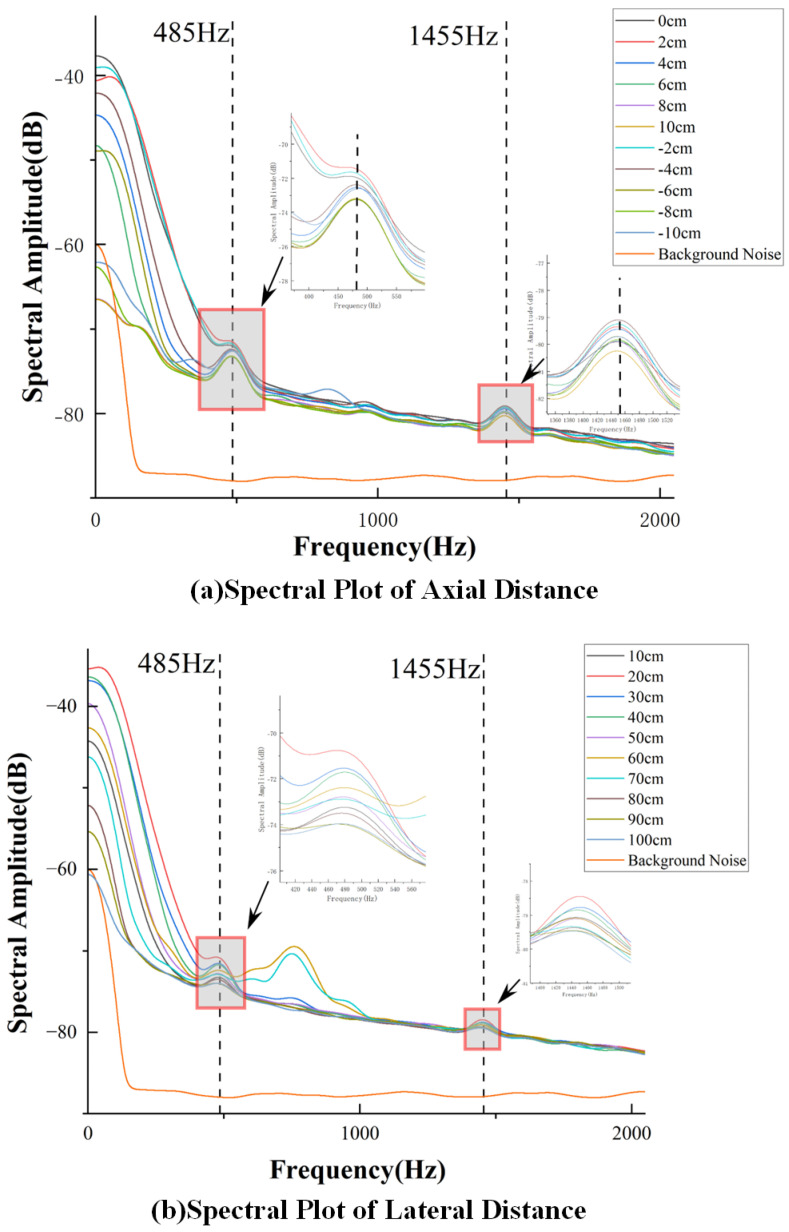
(**a**) Spectral Plot of Different Axial Distances at 960 rpm Operating Condition, (**b**) Spectral Plot of Different Lateral Distances at 960 rpm Operating Condition.

In the previous section, we conducted experiments to measure the time-domain data of the CIWS at different positions. Through Fourier transform (FT), the corresponding spectral data were obtained. By performing FT on each set of time-domain data, the spectral data for all positions were derived. For the set of axial CIWS spectral data at an *x*-axis offset of 0 under an operating condition of 960rpm, as shown in Figure 14a, it can be observed that the overall spectral amplitude initially increases with distance and subsequently decreases as the distance continues to increase. This trend is consistent with the attenuation pattern of the average wake velocity. The effective spectral information is mainly concentrated in the low-frequency range, while the spectral amplitudes at frequencies above 2kHz are nearly identical for different distances, indicating limited research significance in this region.

The background noise remains stable and low in amplitude due to the absence of significant signal content. Notably, the spectrum features two characteristic frequency points at 485Hz and 1455Hz. Observing the spectral data beyond an axial distance of 80cm, as shown in Figure 14a, reveals that the spectral curves tend to converge, suggesting that the sensor’s ability to detect wake information is significantly diminished at axial distances greater than 80cm.

For the set of lateral CIWS spectral data at a *z*-axis offset of 30cm under an PJP operating condition of 960rpm, as shown in Figure 14b, it can be observed that the overall spectral amplitude is highest at a lateral offset of 0cm. As the lateral distance increases, the spectral amplitude decreases. Beyond a lateral offset of 5cm, the spectral curves tend to converge, indicating that the sensor’s ability to detect wake information is significantly weakened for lateral offsets greater than 5cm. Furthermore, the high-frequency portion of the spectrum is found to have limited research significance. Notably, the spectrum also shows two characteristic frequency points at 485Hz and 1455Hz.

### 5.2. Characteristic Frequency Analysis

The characteristic values present in the spectral signal of the CIWS have practical physical significance; these characteristic values correspond to the vertical pulsation frequency of the PJP and its subharmonic frequency. Furthermore, the wake detection range of the CIWS can also be derived from the spectral signal, and the rotational speed of the PJP can be inferred from the spectral data.

The two characteristic frequencies of 485Hz and 1455Hz, observed in Figure 14, are related to the rotation speed of the PJP. According to the previously established inertial reference coordinate system *O*-*xyz* at the rear of the PJP. The number of rotor blades is denoted by $Z_{R}$, and the number of stator blades is denoted by $Z_{S}$. In subsequent texts, the rotor and stator are also represented by *R* and *S*, respectively, with the rotor speed denoted by *N*.The lateral force along the *x*-axis is defined as $F_{x}$, the vertical force along the *y*-axis is defined as $F_{y}$, and the axial force along the *z*-axis is defined as $F_{z}$. $F_{t}$ and $F_{r}$ represent the tangential force and radial force of the blade, respectively. A rotating coordinate system $O-x_{1}y_{1}z_{1}$ is also established, aligned in the same direction as the inertial reference frame O−xyz. The rotational frame of reference has the same angular direction and speed as the rotor This definition is used to analyze the vibration frequency of the lateral force $F_{x}$ of the PJP.

According to Rao [23], the lateral force of the rotor is given by

(9) $$F_{x,R}=-\frac{Z_{R}}{2}\sum_{k=1}^{\infty }B_{r,t,k}\cdot \sin\left(nZ_{R}\theta -\beta_{rk}-\phi_{-}^{+}\right)$$

where

$$\phi_{-}^{+}=\arctan\left(\frac{\mp B_{rk}-B_{tk}\cos\left(\beta_{rk}-\beta_{tk}\right)}{B_{tk}\sin\left(\beta_{rk}-\beta_{tk}\right)}\right),$$

(10) $$B_{r,t,k}=\sqrt{{\left[\mp B_{rk}-B_{tk}\cos\left(\beta_{rk}-\beta_{tk}\right)\right]}^{2}+{\left[B_{tk}\sin\left(\beta_{rk}-\beta_{tk}\right)\right]}^{2}}$$

In the equation, $B_{tk}$ and $B_{rk}$ represent the amplitude of the tangential force and radial force at step *k*, respectively. $\beta_{tk}$ and $\beta_{rk}$ represent the phase angles of the tangential and radial forces, respectively, at step *k*. θ represents the angular position of the reference line of the 0-th rotor.The sign $\mp B_{rk}$ takes the negative sign when $kZ_{S}+1=nZ_{R}$ and the positive sign when $kZ_{S}-1=nZ_{R}$.

The lateral force of the stator and duct is given by

(11) $$F_{x,S}=-\sum_{k=1}^{\infty }\frac{Z_{S}}{2}\left\{B_{r_{1},t_{1},k}\cdot \sin\left(kZ_{R}\theta -\beta_{t_{1},k}-\psi_{-}^{+}\right)\right\}$$

where

$$\psi_{-}^{+}=\arctan\left(\frac{B_{t_{1},k}\pm B_{r_{1},k}\cos\left(\beta_{r_{1},k}-\beta_{t_{1},k}\right)}{\pm B_{r_{1},k}\sin\left(\beta_{r_{1},k}-\beta_{t_{1},k}\right)}\right),$$

(12) $$B_{r_{1},t_{1},k}=\sqrt{{\left[B_{t_{1},k}\pm B_{r_{1},k}\cos\left(\beta_{r_{1},k}-\beta_{t_{1},k}\right)\right]}^{2}+{\left[\pm B_{r_{1},k}\sin\left(\beta_{r_{1},k}-\beta_{t_{1},k}\right)\right]}^{2}}$$

$B_{t_{1}k}$ and $B_{r_{1}k}$ represent the amplitude of the *k*-th order tangential force and radial force, respectively, in the rotating coordinate system *O*-$x_{1}y_{1}z_{1}$. $\beta_{t_{1}k}$ and $\beta_{r_{1}k}$ denote the coordinated phase angles of the tangential and radial forces, respectively, for the *k*-th order.The term $\pm B_{r_{1},k}$ takes a positive sign when $kZ_{S}+1=nZ_{R}$ is satisfied and a negative sign when $kZ_{S}-1=nZ_{R}$ is satisfied.

It is evident that the conditions for the lateral force of both the stator and rotor are the same, i.e., $kZ_{S}\pm 1=nZ_{R}$, and they share the same form of expression. Therefore, by adding the lateral forces of the stator and rotor, the total lateral force of the propeller can be written as

(13) $$F_{x}=\sum_{k=1}^{\infty }B_{k}\cdot \sin\left(nZ_{R}\theta +\xi \right)$$

The duct can be considered part of the stator; thus, the derivation of the pulsation frequency of the stator’s unsteady force also includes the lateral force component of the duct.

Let t=0 denote the initial angular position of the 0th rotor as zero, then we have θ=2πNt. Substituting into Equation (11) yields the vibration frequency of the total lateral force of the propeller:

(14) $$f_{x,kn}=\frac{2nZ_{R}N\pi }{2\pi }=nZ_{R}N$$

The conditions $kZ_{S}+1=nZ_{R}$ or $kZ_{S}-1=nZ_{R}$ must be strictly satisfied, where *k*, *n*, $Z_{S}$, and $Z_{R}$ are all positive integers.

For the PJP selected in this study, the number of rotor blades is $Z_{R}=9$, and the number of stator blades is $Z_{S}=7$. The actual rotation speed of 960rpm corresponds to a rotor speed of N=16rps. When k=10 and n=13, the condition $kZ_{S}+1=nZ_{R}$ is satisfied, resulting in $f_{x,kn}=1455\;\mathrm{Hz}$.

It can be observed in Figure 14 that a characteristic value exists at 1455Hz, as well as a subharmonic frequency of $f_{x,kn}$ at 485Hz (which is 1/3 of $f_{x,kn}$). However, the spectral amplitude at $f_{x,kn}$ is relatively low, and the degree of concentration is not as distinct as at 485Hz. Therefore, 485Hz is selected as the characteristic frequency for further analysis.

### 5.3. Detection Data and Flow Field Fitting

By substituting the positional information of the CIWS data into the wake velocity decay model in Section 2, the average velocity value corresponding to that position can be calculated. The spectral amplitude data of the sensor at different offset distances along the *z*-axis (axial) under the 960 rpm condition were preprocessed and fitted to the corresponding average velocity. From Figure 14, it can be observed that the data at 485 Hz exhibit greater dispersion compared to those at 1455Hz, and there is an approximately linear relationship between the spectral amplitude and the average velocity. Therefore, the spectral amplitude at the characteristic frequency of 485 Hz was chosen for linear fitting with the corresponding average velocity using the following equation:

(15) A=kv+b

where *A* is the spectral amplitude, and *v* is the average velocity.

**Figure 15:**
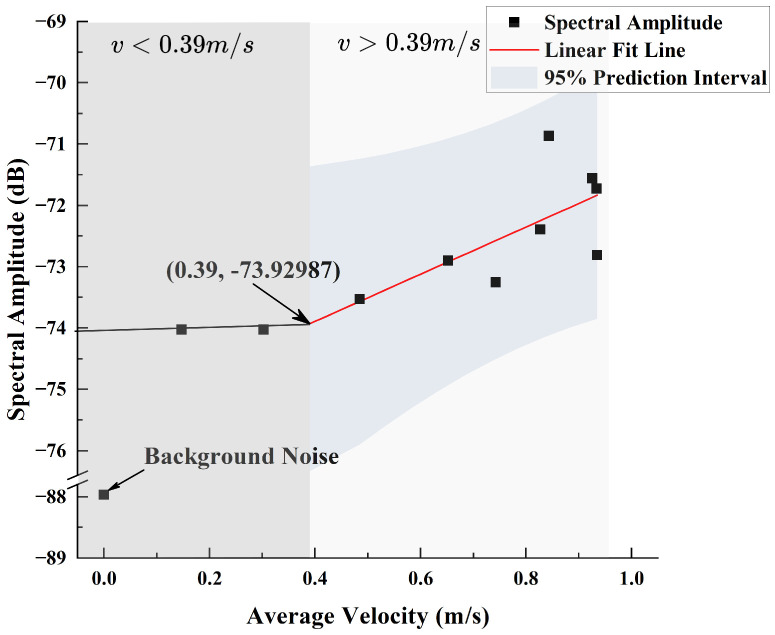
Fitting Performance Between Axial Spectral Amplitude at 485 Hz and Flow Velocity.

Since the spectral data beyond an axial distance of 80 cm tend to be similar, this portion of the data negatively impacts the fitting process and was therefore excluded. The remaining valid data were fitted based on Equation (15), yielding k=3.85 and b=−75.43, as shown in Figure 15. By intersecting the fitted line of the valid data with that of the excluded data, the intersection point (0.39,−73.92987) can be obtained. This indicates that when the average velocity is less than 0.39 m/s, the spectral amplitude of the sensor no longer changes with the velocity.

**Figure 16:**
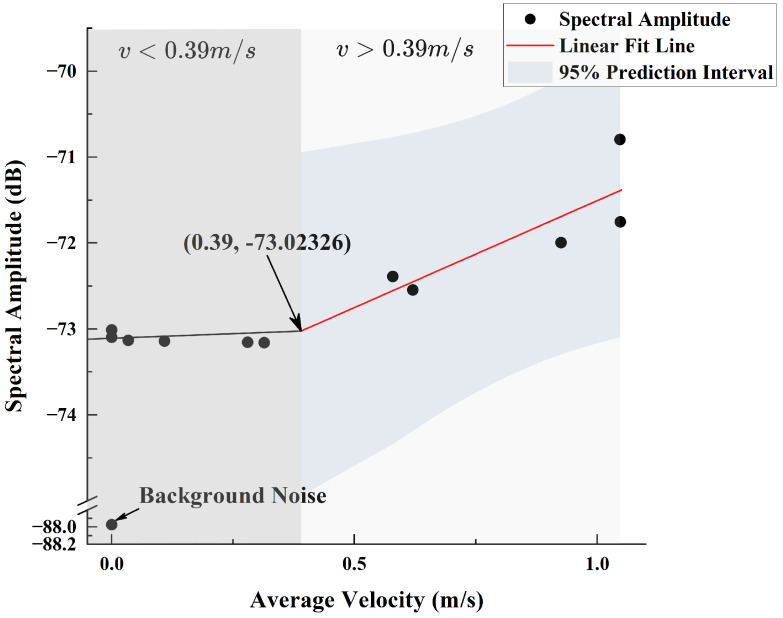
Fitting Performance Between Lateral Spectral Amplitude at 485 Hz and Flow Velocity.

The same method was used to investigate the fitting relationship between the spectral amplitude and average velocity at different *x*-axis (lateral) offset distances under the 960 rpm condition. The spectral data beyond a lateral offset of 4 cm tended to converge, so these data were excluded. The remaining valid data were fitted based on Equation (15), yielding k=2.49 and b=−73.99, as shown in Figure 16. Similarly, by intersecting the line fitted to the excluded data with the linear fitting line, the intersection point (0.39,−73.02326) was obtained, indicating that the critical velocity value is also 0.39 m/s.

From Figure 15 and Figure 16, it can be seen that whether along the axial or lateral direction of the propeller, when the flow velocity is less than 0.39 m/s, the CIWS can still detect wake information, but the spectral amplitude does not change with distance. Therefore, the range within which the wake velocity information can be inferred from the CIWS data is smaller than the range in which the sensor can detect propeller noise.

**Figure 17:**
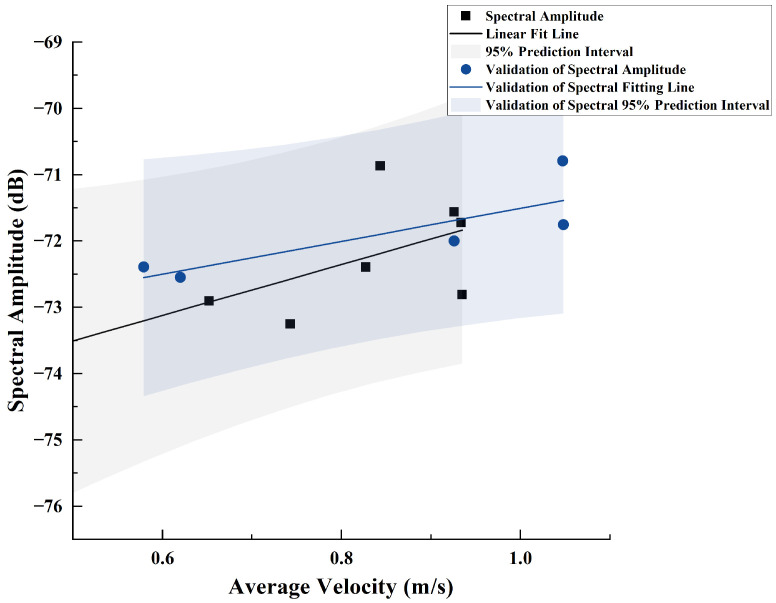
Explanatory Power of Axial Fitting Results for Lateral Group Data.

Substituting the fitting result of the lateral spectral amplitude at 485Hz with respect to the flow velocity into the axial fitting result yields the relationship shown in Figure 17. From Figure 17, it can be observed that the overlap of the 95% prediction interval is relatively high, suggesting that the spectral amplitude detected by the sensor is primarily influenced by the flow velocity at that position and shows low correlation to the relative flow direction.

### 5.4. Wake Detection Range

**Figure 18:**
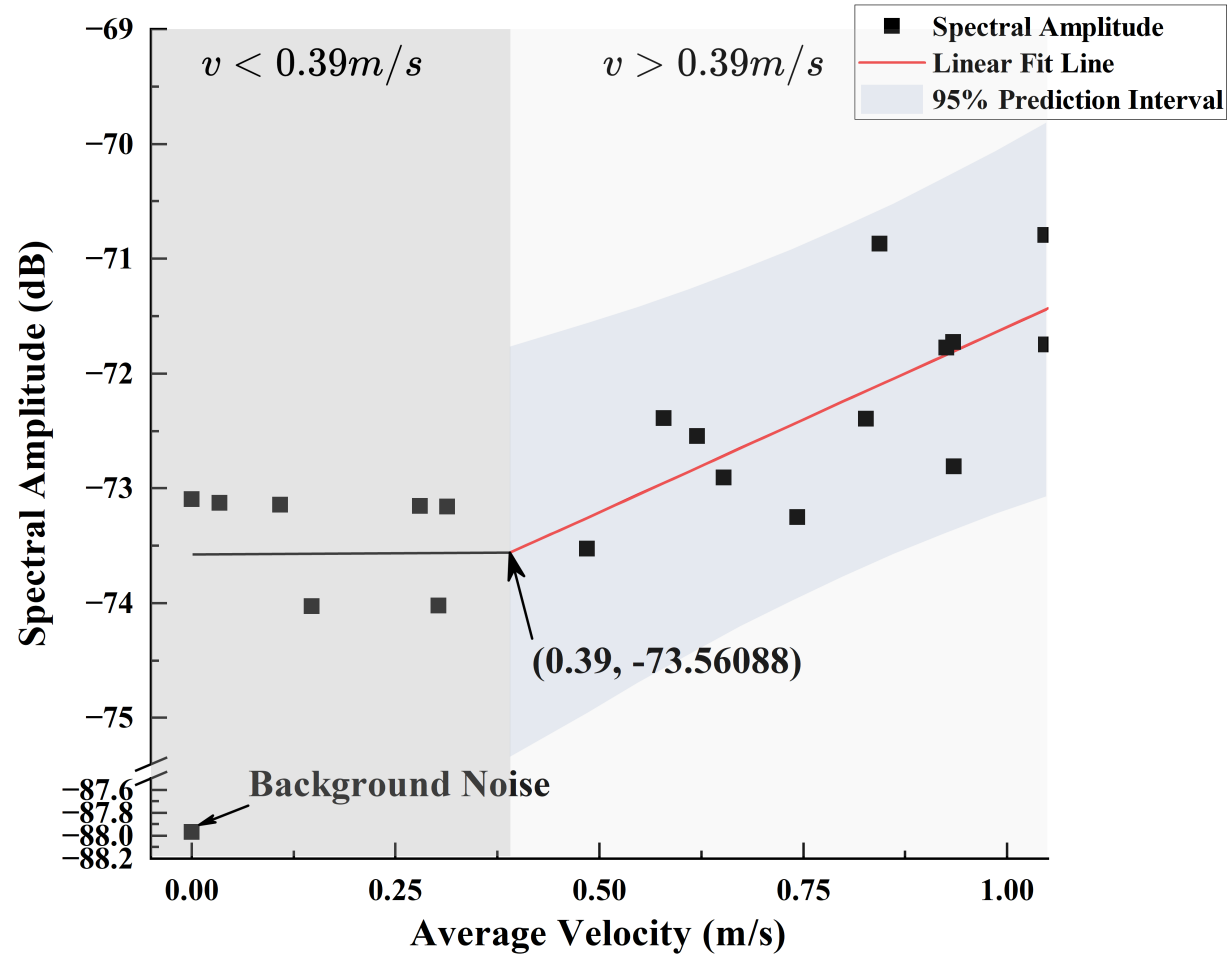
Fitting Results Between Mean Velocity and Spectral Amplitude at 485 Hz.

By combining the data from Figure 15 and Figure 16, we can obtain Figure 18. From the figure, it can be seen that the minimum flow velocity detection threshold for the sensor is 0.39 m/s, which is consistent with the detection range conclusions from Figure 15 and Figure 16. For the detection data greater than 0.39 m/s, fitting using Equation (15) yields k=3.23 and b=−74.82.

**Figure 19:**
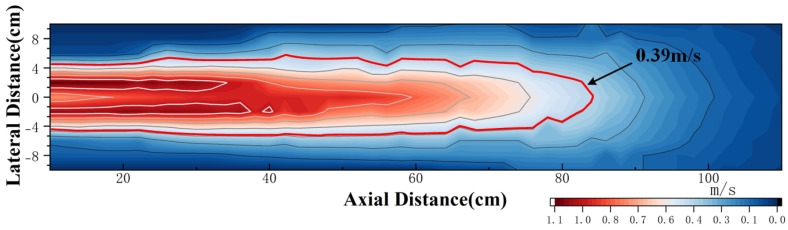
Effective Detection Range of Average Wake Velocity for AUV.

By substituting the threshold value of 0.39 m/s, which can be used to infer the wake velocity from the spectral amplitude of the CIWS, into the wake velocity decay model, the curve shown in Figure 19 is obtained. The area within this curve represents the range within where the CIWS can detect wake velocity information. From the figure, it can be seen that the velocity detection range of the sensor is ±5 cm in the *x*-axis direction and 85 cm in the *z*-axis direction.

**Figure 20:**
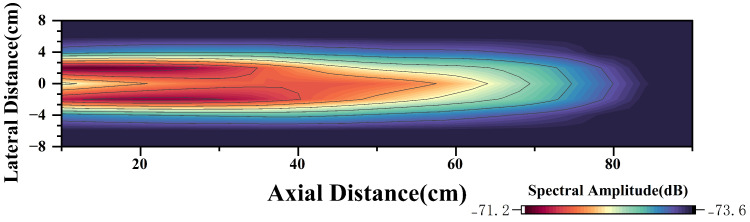
Contour Plot of Signal Amplitude Variation with Position.

Further overlaying the relationship between the spectral amplitude of the CIWS and the wake velocity into Figure 20 yields the corresponding model for the sensor spectral amplitude and position, as shown in Figure 20. Using this model, underwater vehicles such as AUVs equipped with CIWSs can estimate their possible position relative to the PJP based on the sensor’s spectral data. With the method above, by matching the detected velocity and the trend of position change within the wake field, the specific position of the sensor within the wake model can be established.

**Figure 21:**
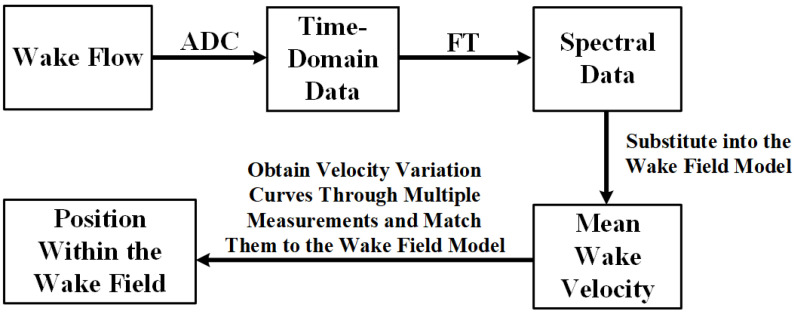
Workflow for AUV Wake Detection and Positioning.

The specific workflow for AUV wake detection and positioning is illustrated in Figure 21. Once the wake enters the CIWS, it undergoes analog-to-digital conversion (ADC) to obtain time-domain signal data. Subsequently, the time-domain data are transformed into spectral data through Fourier transform (FT). Using the linear relationship between the spectral amplitude and the mean wake velocity, the mean velocity at the current position can be determined. By performing multiple measurements, the motion curve and the corresponding velocities can be matched within the wake field model to estimate the current relative position in the wake field. This process requires further integration with the AUV navigation and control system for practical applications.

## 6. Conclusions

This paper focuses on the feasibility and effectiveness of using CIWS for detecting and tracking AUV wakes. By analyzing the principles of CIWS, conducting experiments and analyzing the experimental data, the following findings and conclusions have been achieved:

First, the feasibility of AUV wake detection using CIWS was investigated based on the working principles of cilium. By analyzing the biomechanical model of the cilium, it was found that four forces are involved during its motion: $F_{m}$, $F_{k}$, $F_{u}$, and $F_{d}$. Spectral analysis of the mechanical model revealed that the ratio of hemispherical displacement amplitude $Y_{0}\left(f\right)$ to the external flow velocity amplitude $V_{0}\left(f\right)$ exhibits a linear relationship in the low-frequency range, providing theoretical support for subsequent experiments.

Second, using a flow velocity sensor, the flow velocities at different positions behind the PJP in a moored state were measured. Then, an average velocity model of the AUV wake flow field was established.

Third, the CIWS was used to measure different positions within the PJP wake range, obtaining time-domain and frequency-domain data of the CIWS at various positions. Combined with the previously established flow field model, the time-domain and frequency-domain data corresponding to different average velocities were obtained.

Fourth, spectral analysis of the CIWS data revealed the presence of characteristic frequencies associated with the lateral pulsation force of the PJP, specifically at 1455Hz and its subharmonic frequency at 485Hz. The flow velocity data and the spectral amplitude at 485Hz for the corresponding CIWS position were linearly fitted, yielding a threshold velocity of 0.39m/s, which could be utilized for position estimation. Further analysis presented a flow field range for estimating the relative position: ±5 cm along the *x*-axis and 85cm along the *z*-axis. Additionally, a linear relationship was established between the spectral amplitude *A* and the average velocity *v* within this flow field range. Based on this relationship and the propeller flow field model, it is possible to estimate the relative position of the sensor with respect to the propeller using the spectral amplitude detected by the CIWS.

In summary, with experimental and data analysis, this paper demonstrates the effectiveness of CIWS in detecting wakes of underwater vehicles, providing a novel method for underwater target detection and positioning. This research establishes a theoretical and technical foundation for underwater vehicle localization and tracking based on wake detection. The proposed method can be applied to underwater missions such as swarm formation of AUVs or detection and tracking of non-cooperative targets.

For future research to meet the demands of underwater target detection and wake tracking, the following work can be conducted based on this study: (1) Integrating CIWS onto dynamic platforms such as AUVs to assess the impact of the platform’s own navigation and vibrations; (2) Conducting dual-AUV wake detection experiments to evaluate the effectiveness of detecting the wake of another sailing AUV by a navigating AUV with CIWS; (3) Conducting dual-AUV navigation experiments to explore AUV navigation and tracking control methods based on underwater target wake detection and positioning with CIWS.

## Figures and Tables

**Table 1:** The specific parameters of the CIWS.

Cilium Structure	Diameter	Length	Operating Voltage
Crossbeam	24 mm	160 mm	±5 V

**Table 2:** The specific parameters of the PJP.

Stator Position	Number of Rotor Blades	Number of Stator Blades	Shroud Inner Diameter	Central Shaft Diameter	Supply Voltage	Set Rotational Speed	Actual Rotational Speed
Aft Stator	Seven Blades	Nine Blades	80 mm	30 mm	24 V	1000 rpm	960 rpm

## Data Availability

Access to the data will be considered upon request by the authors.

## Abbreviations

The following abbreviations are used in this manuscript:

AUV	Autonomous underwater vehicle
CIWS	Cilium-inspired wake sensor
CN	Canal neuromasts
PJP	Pump-jet propeller
ADC	Analog-to-digital conversion
RMS	Root mean square
FT	Fourier transform

## References

[1] Zhang W., Wang N.X., Wei S., Du X., Yan Z. (2020). Overview of unmanned underwater vehicle swarm development status and key technologies. J. Harbin Eng. Univ..

[2] Zhao Z., Hu Q., Feng H., Feng X., Su W. (2022). A cooperative hunting method for multi-AUV swarm in underwater weak information environment with obstacles. J. Mar. Sci. Eng..

[3] Jiang B., Du J., Jiang C., Han Z., Debbah M. (2023). Underwater searching and multi-round data collection via AUV swarms: An energy-efficient AoI-aware MAPPO approach. IEEE Internet Things J..

[4] Ghafoor H., Noh Y. (2019). An overview of next-generation underwater target detection and tracking: An integrated underwater architecture. IEEE Access.

[5] Wei Q., Yang Y., Zhou X., Hu Z., Li Y., Fan C., Zheng Q., Wang Z. (2024). Enhancing Inter-AUV Perception: Adaptive 6-DOF Pose Estimation with Synthetic Images for AUV Swarm Sensing. Drones.

[6] Wei Q., Yang Y., Zhou X., Fan C., Zheng Q., Hu Z. (2023). Localization method for underwater robot swarms based on enhanced visual markers. Electronics.

[7] Wang Q., He B., Zhang Y., Yu F., Huang X., Yang R. (2023). An autonomous cooperative system of multi-AUV for underwater targets detection and localization. Eng. Appl. Artif. Intell..

[8] Lodovisi C., Loreti P., Bracciale L., Betti S. (2018). Performance analysis of hybrid optical–acoustic AUV swarms for marine monitoring. Future Internet.

[9] Zhou Q., Ji B., Wei Y., Hu B., Gao Y., Xu Q., Zhou J., Zhou B. (2019). A bio-inspired cilia array as the dielectric layer for flexible capacitive pressure sensors with high sensitivity and a broad detection range. J. Mater. Chem. A.

[10] Zhou Z.G., Liu Z.W. (2008). Biomimetic cilia based on MEMS technology. J. Bionic Eng..

[11] Astreinidi Blandin A., Bernardeschi I., Beccai L. (2018). Biomechanics in soft mechanical sensing: From natural case studies to the artificial world. Biomimetics.

[12] van Netten S.M. (1991). Hydrodynamics of the excitation of the cupula in the fish canal lateral line. J. Acoust. Soc. Am..

[13] Van Netten S.M. (2006). Hydrodynamic detection by cupulae in a lateral line canal: Functional relations between physics and physiology. Biol. Cybern..

[14] Sengupta D., Trap D., Kottapalli A.G. (2020). Piezoresistive carbon nanofiber-based cilia-inspired flow sensor. Nanomaterials.

[15] Qiao Q., Kong X., Wu S., Liu G., Zhang G., Yang H., Zhang W., Yang Y., Jia L., He C. (2023). A bio-inspired MEMS wake detector for AUV tracking and coordinated formation. Remote Sens..

[16] Kim J. (2024). Wake-Responsive AUV Guidance Assisted by Passive Sonar Measurements. J. Mar. Sci. Eng..

[17] Kim D.H., Kim N., Cho H., Kim S.Y. A guidance logic development for wake homing guidance system (ICCAS 2014). Proceedings of the 2014 14th International Conference on Control, Automation and Systems (ICCAS 2014).

[18] Liu L., Su S., Huang Z. (2010). Design of Torpedo Wake Homing System for Higher Trajectory Accuracy. J. Unmanned Undersea Syst..

[19] Basohbat Novinzadeh A.R., Asadi Matak M. (2017). Design of stable nonlinear guidance of an underwater vehicle in the ship wake via estimated path by particle filter. Modares Mech. Eng..

[20] Shizhe T. (2014). Underwater artificial lateral line flow sensors. Microsyst. Technol..

[21] Qiao Q. (2023). Design and Implementation of a Bio-Inspired MEMS Wake Sensor for UUV. Master’s Thesis.

[22] Yang Y., Zhou X. (2022). Research on High-precision Unmanned Underwater Vehicles Team Formation without Communication Based on Visual Positioning Technology. Digit. Ocean. Underw. Warf..

[23] Rao Z.Q. (2012). Numerical Simulation of Hydrodynamical Performance of Pump Jet Propulsor. Master’s Thesis.

